# On the Use of a Convolutional Block Attention Module in Deep Learning-Based Human Activity Recognition with Motion Sensors

**DOI:** 10.3390/diagnostics13111861

**Published:** 2023-05-26

**Authors:** Sumeyye Agac, Ozlem Durmaz Incel

**Affiliations:** Department of Computer Engineering, Bogazici University, Istanbul 34342, Turkey

**Keywords:** human activity recognition, motion sensors, convolutional neural networks, hybrid deep models, attention mechanism, channel attention, spatial attention

## Abstract

Sensor-based human activity recognition with wearable devices has captured the attention of researchers in the last decade. The possibility of collecting large sets of data from various sensors in different body parts, automatic feature extraction, and aiming to recognize more complex activities have led to a rapid increase in the use of deep learning models in the field. More recently, using attention-based models for dynamically fine-tuning the model features and, in turn, improving the model performance has been investigated. However, the impact of using channel, spatial, or combined attention methods of the *convolutional block attention module (CBAM)* on the high-performing *DeepConvLSTM* model, a hybrid model proposed for sensor-based human activity recognition, has yet to be studied. Additionally, since wearables have limited resources, analysing the parameter requirements of attention modules can serve as an indicator for optimizing resource consumption. In this study, we explored the performance of CBAM on the DeepConvLSTM architecture both in terms of recognition performance and the number of additional parameters required by attention modules. In this direction, the effect of channel and spatial attention, individually and in combination, were examined. To evaluate the model performance, the Pamap2 dataset containing 12 daily activities and the Opportunity dataset with its 18 micro activities were utilized. The results showed that the performance for Opportunity increased from 0.74 to 0.77 in the macro f1-score owing to spatial attention, while for Pamap2, the performance increased from 0.95 to 0.96 owing to the channel attention applied to DeepConvLSTM with a negligible number of additional parameters. Moreover, when the activity-based results were analysed, it was observed that the attention mechanism increased the performance of the activities with the worst performance in the baseline model without attention. We present a comparison with related studies that use the same datasets and show that we could achieve higher scores on both datasets by combining CBAM and DeepConvLSTM.

## 1. Introduction

Mobile and wearable device diversity and usage rates are expanding due to technical advancements. These devices have several integrated sensors, particularly motion sensors, that can be used to track the user’s movements. Sensor-based human activity recognition (HAR) [1,2,3] is a popular research topic since mobile and wearable devices integrated with various sensors, such as accelerometers, gyroscopes, and heart rate sensors, generate vast amounts of data about users’ behaviours, activities, and context. However, there is still much room for advancement given the challenges, including collecting labelled data for model training, the variety of human activities, changing human behaviours, and dataset imbalance.

One challenge involves the positions where the device is carried/worn on the body. Sensor readings coming from different body parts with the changing activities differ. Several studies have only used a single body-location sensor(s) for HAR [4,5,6]. However, a single sensor at a specific body location, such as an inertial measurement unit (IMU) on the user’s chest, may only capture local body movements, resulting in low recognition accuracy, especially for complex activities, such as rowing. Moreover, the activity set may include many activities of various types that impact various body parts, even if the activity is as straightforward as sitting. As a result, HAR needs to employ/fuse data gathered from several body areas to recognize the activities effectively in terms of accuracy and reliability [7]. However, not all sensors may equally contribute to the recognition performance, and it is important to assign specific importance to data coming from different positions for different activities.

Sensor data collected from different body parts is time-series data; hence, HAR is modelled as a multivariate time-series classification problem. Often, sensor signals are segmented into windows to capture the temporal information in the sequence of activities. Deciding on the optimal window size is one of the challenges since it impacts recognition performance. Many different classification algorithms have been applied in the HAR domain; in particular, shallow models have been extensively studied [1]. They achieve acceptable performances on smaller datasets. However, they require extensive feature engineering to achieve a good level of recognition. By virtue of its multilayered structure, deep learning enables automatic feature extraction and increases the accuracy and robustness of the models [3]. Its use is widespread in the recent sensor-based HAR studies [8,9,10,11,12,13,14] and also has been presented as one of the future directions and/or challenges of HAR in recent surveys [15,16,17,18].

Particularly, sequential deep learning models, such as LSTM, can help in modelling the temporal information in time-series signals and overcome the challenge of deciding on window size. However, as mentioned in [19], an event that occurs far away in time may not impact the current events as much as a recent one.

Attention mechanism [20] is an emerging method for dynamically fine-tuning neural network features to improve the model’s performance. It has recently been widely used across various research domains, such as natural language processing [21] and computer vision [22]. From a sensor-based HAR perspective, the attention mechanism can help the deep model to learn the most contributing signal better to distinguish human activities [17] by allowing the deep network to dynamically adjust its focus for target activities [23]. Given an appropriate attention design, the network can automatically amplify the influence of informative features and suppress unrelated noise.

There are a few recent studies that have investigated the incorporation of some attention mechanisms in deep models for HAR [24,25,26,27,28,29] models. However, there is still room for improvement in exploring the attention methods proposed in other domains, such as computer vision, into deep models, particularly proposed for the sensor-based HAR domain, such as DeepConvLSTM [30]. In this paper, we examine the effect of the convolutional block attention module (CBAM) [31] on the DeepConvLSTM benchmark. CBAM is an attention mechanism that boosts performance by enhancing informative channels and important regions of intermediate features. We can overcome both the challenge of treating data from body parts and the windowing aspect. Additionally, in [31], authors emphasize that the CBAM is a lightweight module which can be added to any convolutional neural network (CNN) with negligible overheads, which makes it a suitable approach for working with resource-constrained mobile and wearable devices.

In order to evaluate the performance of using CBAM in HAR, we used two datasets: Pamap2 [32] and Opportunity [33].These datasets are extensively used in the sensor-based HAR domain [3], particularly in studies focusing on attention [19,27,29,34,35,36]. Additionally, they are collected from different body positions, and activity sets are diverse. Pamap2 contains 12 daily activities, and the Opportunity dataset includes 18 micro activities (such as opening a drawer). CBAM has two submodules: the channel attention (CHatt) module and the spatial attention (SPatt) module. These modules can be used in different combinations: both in parallel, one of them, both with SPatt first, and both with CHatt first. We investigated CHatt alone, SPatt alone, CHatt first, and then SPatt first. We investigated the impact of channel attention by using various reduction ratios and spatial attention by using various kernel sizes at different depths (such as after layer 1, layer 2, etc.). The results showed that the performance for the Opportunity dataset increased from 0.74 to 0.77 for the macro f1-score with spatial attention, while for the Pamap2 dataset, the performance increased from 0.95 to 0.96 using channel attention. When the activity-based results were analysed, we observed that the attention mechanism particularly increased the performance of the activities (such as descending stairs) that had worse performance in the base model without attention. The main highlights of this paper are as follows:

- Although the effect of using attention with some deep learning architectures has been investigated in related studies, we are interested in exploring how an already good-performing deep model, DeepConvLSTM, can benefit from channel, spatial, or both attention methods.
- We performed an extensive set of experiments to explore how different reduction ratios and kernel sizes of attention and the application of attention at different depths of the deep architecture impact the performance of recognition on two different datasets where multiple body positions were involved with a large set of activities. In Section 5, we present a comparison with studies that utilize attention on the same datasets and show that we could achieve higher scores using the combination of CBAM and DeepConvLSTM.
- We also found that adding attention did not significantly increase the number of additional model parameters. Although we did not run these models on a mobile or wearable device in this work, having less complex models is important for future work when these models are ported to mobile or edge devices where computational resources are limited compared to a server or a cloud environment [37].

The remainder of the paper is organised as follows: We present the related studies focusing on attention on sensor-based HAR in Section 2. The methodology is explained in Section 3. The performance evaluation of the recognition systems is presented in Section 4. Finally, we present the discussion in Section 5 and the conclusions in Section 6.

## 2. Related Work

Humans divide their attention by highlighting interesting information and suppressing irrelevant and potentially confusing ones while performing complex tasks. Model attention in deep neural networks inspired by humans’ perceptions is proposed in [20]. As mentioned, using attention-based models has recently been explored in different research domains, such as natural language processing [21] and computer vision [22].

To the best of our knowledge, few recent attempts have focused on using an attention mechanism in the sensor-based HAR domain. Recently, in [29], authors proposed attention-based long short-term memory (LSTM) to address the dependencies of multimodal sensor signals in spatial and temporal domains for HAR. They were particularly interested in which sensor modalities are placed on different body parts (called where) to focus on and which part of the time-series (when) to focus on simultaneously. Considering the Pamap2 dataset [32], the average f1-score obtained using LSTM without attention was 0.75, which was increased to 0.90 with use of their attention-based LSTM model. Similarly, in [34], the authors propose a multimodal sensing HAR model using spatial and temporal attention. This study combined attention mechanisms into a gated recurrent unit (GRU) subnet to improve the recognition performance of recurrent networks. Their system achieved 0.89 recognition performance for the Pamap2 dataset. However, CNN architecture has a more powerful feature extraction and classification capability than recurrent neural networks.

In [38], the authors propose a self-attention deep learning framework in order to fuse heterogeneous and time-series information, respectively. The mean of all feature vectors is used as the query to estimate local features’ attention weight from each sensor. In [36], authors obtained insight from the deep neural networks where the lower layers have features local to the input and general to the task, while higher layers have features global to the input and specific to the current activity class [39]. Their framework has two attention modules: global position attention and global modality attention.

The authors of [27] were the first to propose a framework blending temporal and channel attention (called dual attention) on convolution networks for multimodal HAR purposes. They used a CNN network with an attention module inspired by the CBAM module [31]. The model extracts channel attention using pooling layers to combine features along the temporal dimension. They also investigated the impact of adding residual connections to the architecture. The proposed model achieved better performances on commonly used multimodal HAR datasets, such as Pamap2 [32] and Opportunity [33]. More clearly, considering Opportunity, their standard CNN achieved an accuracy of 0.78 (with 1.15 M parameters), while the attention-based CNN achieved an accuracy of 0.80. By using an attention-based residual network, they achieved a recognition accuracy of 0.83, but the number of model parameters increased to 1.57 M, which is not preferable regarding resource consumption. For Pamap2, their attention-based model improved the accuracy of CNN from 0.78 (with 2.73 M parameters) to 0.92 (with 2.75 M parameters). Overall, applying only dual attention to the CNN yielded a 0.03 and 0.01 increase in accuracy for Opportunity and Pamap2 datasets, respectively. In our study, by using DeepConvLSTM with CBAM modules, we achieved a performance improvement in the macro f1-score of 0.03 and 0.01 for Opportunity and Pamap2, respectively. Moreover, our models consumed fewer resources (0.8 2M parameters for Opportunity and 1.42 M for Pamap2) since the number of parameters is low compared to that of dual attention-based CNN models.

In another study [24], the authors propose an attention-based sensor fusion using IMUs. They use image representations of time windows as input to the system. After data representation, they extract features separately for each body location using convolution operations with different kernel sizes (1 × 3, 3 × 3, and 5 × 5). They concatenate features and then investigate sensor-wise attentions to use later in classification.

The most recent study [25], exploits the importance of the cross-domain interactions of sensor signals which are temporal–spatial, temporal–channel, and channel–spatial interactions. Therefore, they include three attention branches in parallel to a plain CNN architecture and propose a cross-domain attention model for sensor-based HAR. Each branch applies an attention module similar to that presented in [31].

Although innovations and advantages brought by deep learning algorithms are desirable for use in the field of HAR to increase recognition performance, resource consumption is a challenging issue in mobile and wearable devices. These devices are especially limited in terms of battery and memory, and even running the algorithms on these devices in the inference mode is a problem that requires attention. The number of additional parameters brought by applying attention is generally less, as in the case of the bottleneck attention module [40] and CBAM [31], compared to the number of parameters required in overall deep architecture. In [31] especially, the authors emphasize that the CBAM is a lightweight module which can be added to any CNN with negligible overhead.

The authors of [41] introduced a two-stage end-to-end CNN model for human activity recognition. The model utilizes a deep CNN as a feature extractor and a shallow CNN as a classifier to learn informative features from input sensor data and classify them into different activity categories. The study did not include any attention mechanism, but the model was designed to be computationally efficient. Therefore, the proposed model is suitable for real-time predictions and can be used on resource-constrained devices.

In [19], the authors add attention to the state-of-the-art deep HAR model DeepConvLSTM [30]. They investigated the impact of attention by applying the module on top of the LSTM layers of DeepConvLSTM model to improve the performance of the models. They evaluated their approach o then Pamap2 [32], Opportunity [33]. and Skoda [42] datasets. The proposed system increased the f1-score from 0.672 to 0.707 for Opportunity, from 0.748 to 0.875 for Pamap2, and from 0.912 to 0.913 for Skoda. Their attention module focuses on the temporal part (LSTM), and any attention to convolutional layers of DeepConvLSTM is not considered, unlike in our work.

Researchers have begun to use CNN-based architectures frequently in the sensor-based HAR domain because of the considerable resource consumption of time-series models (e.g., RNN) and the quick/efficient development of new CNN-based approaches. Subsequently, new models have emerged, starting with convolutional layers, which have an effective feature extraction capability and can thus incorporate temporal dependencies with subsequent LSTM layers as in the case of DeepConvLSTM architecture. To the best of our knowledge, only one study has employed attention on DeepConvLSTM [19] with a focus on adding attention on top of LSTM layers to better weigh the temporal context. However, the effect of CBAM, which was originally proposed for the computer vision domain, on the sensor-based HAR benchmark DeepConvLSTM, has not yet been examined. This architecture is a suitable candidate for implementing CBAM, as it contains convolutional layers. As a matter of fact, it is mentioned in the CBAM study that this attention mechanism can be easily applied to any CNN structure. Since the related attention method does not require many additional parameters, the CBAM method is a suitable candidate for the sensor-based HAR domain where resource-constrained devices are targeted. For this reason, in this study, we examined the effect of the CBAM attention method on the DeepConvLSTM benchmark. By using spatial attention applied to the third convolutional layer of DeepConvLSTM with a kernel size of 5, we increased the f1-score of the Opportunity dataset from 0.74 to 0.77. Moreover, for Pamap2, the channel attention applied to the fourth convolutional layer of DeepConvLSTM with a reduction ratio of 2 achieved a performance improvement from 0.95 to 0.96. We present a comparison with related studies in Section 5 that use the same datasets and show that we could achieve higher scores on both datasets by using CBAM with DeepConvLSTM.

## 3. Methodology

**Figure 1:**
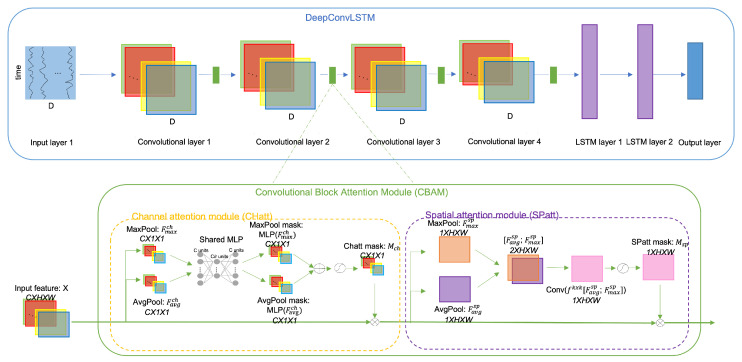
DeepConvLSTM architecture with the Convolutional Block Attention Module.

In this section, we explain the building blocks of our methodology. First, we summarize the CBAM module and then the details of the DeepConvLSTM model. The integration details of these blocks are presented in Figure 1, and finally, we explain the details of the datasets along with implementation details.

### 3.1. Convolutional Block Attention Module

In [31], authors proposed the Convolutional Block Attention Module (CBAM), an attention mechanism that boosts performance by enhancing informative channels and important regions of intermediate features. The main study evaluates the impact of CBAM using datasets commonly used in computer vision research, such as ImageNet [43] and CIFAR-10 [44]. However, they did not utilize sensor data in these experiments. In addition to the possibility of increasing the performance, it is a promising candidate for sensor-based event recognition models with a convolutional layer in it, as the number of parameters required by attention modules is very few (negligible), and the module can be easily applied to any convolutional layer. CBAM comprises two sub-modules: channel attention (*CHatt*) module and spatial attention (*SPatt*) module. As the names suggest, the modules are designed to be applied after convolutional layers.

***CHatt*** **module** uses maximum pooling (sharper effect) and average pooling (smoothing effect) in spatial dimension to the input feature, a multilayer perceptron (MLP) mapped by reduction ratio (*r*), and then a sigmoid activation is applied. The reduction ratio is the key parameter that controls the degree of dimensionality reduction to create a trade-off between computational efficiency and attention accuracy through a shared MLP module in the channel attention mechanism. A smaller reduction ratio can increase the expressive capacity of the channel attention mechanism but with the cost of higher computational complexity and vice versa for a larger reduction ratio. Depending on the specific application, the reduction ratio should be carefully tuned to achieve the best balance between attention performance and computational efficiency. More precisely, to compute channel attention map $M_{ch}$∈${}^{CX1X1}$ given an input feature *X*∈${}^{CXHXW}$, first of all, two vectors which are maximum and average pooling in spatial dimension are computed using input feature *X*: $F_{avg}^{ch}$ and $F_{max}^{ch}$∈${}^{CX1X1}$. Then, these vectors are passed one by one as input to the shared MLP, which has *C* neurons in the input layer, C/r neurons in the hidden layer and *C* neurons in the output layer. After two output vectors are obtained from MLP, these vectors are merged using element-wise summation. Then, a sigmoid (σ) activation layer is applied in order to map values within the range of 0 and 1. Finally, all elements of each channel in *X* are multiplied by its corresponding channel attention value. The steps used to compute the channel attention map are as follows:

(1) $$F_{avg}^{ch}=GlobalAvgPool^{sp}\left(X\right)$$

(2) $$F_{max}^{ch}=GlobalMaxPool^{sp}\left(X\right)$$

(3) $$M_{ch}\left(X\right)=\sigma \left(MLP\left(F_{avg}^{ch}\right)+MLP\left(F_{max}^{ch}\right)\right)$$

***SPatt*** **module** consists of three consecutive operations. First, two tensors, $F_{avg}^{sp}$ and $F_{max}^{sp}$∈${}^{1XHXW}$, are computed using maximum and average pooling across channels of input feature *X*. Second, two tensors are concatenated and passed as an input to the convolution layer (Conv(.)) with a kernel size of *k*x*k* to generate one channel feature map (∈${}^{1XHXW}$). Third, the sigmoid activation layer is applied to the output in order to obtain the final spatial attention mask. Finally, all the feature maps in *X* are multiplied element-wise by the spatial attention mask generated. The following equations are applied to compute the spatial attention mask:

(4) $$F_{avg}^{sp}=GlobalAvgPool^{ch}\left(X\right)$$

(5) $$F_{max}^{sp}=GlobalMaxPool^{ch}\left(X\right)$$

(6) $$M_{sp}\left(X\right)=\sigma \left(Conv\left(f^{kxk}\left[F_{avg}^{sp};F_{max}^{sp}\right]\right)\right)$$

Given an input feature *X*, the overall CBAM is as follows:

(7) $$X^{\prime }=M_{ch}\left(X\right)$$

(8) $$X^{\prime \prime }=M_{sp}\left(X^{\prime }\right)$$

CBAM uses both spatial and cross-channel relationships of features by successively combining channel and spatial attention. To be more precise, it highlights helpful channels and strengthens local regions that are informative. The CBAM has a lightweight design. *CHatt* module requires 2∗C∗(C/r)+C+(C/r) parameters to learn in shared MLP, and *SPatt* module requires k∗k∗2 parameters with *k* as the kernel size of the convolutional layer. From this point of view, it is understandable that the improvements made possible by CBAM are not related to the model’s increased capacity but to efficient feature refining. It should be noted that *r* for *CHatt* and *k* for *SPatt* module are the only parameters that can be selected experimentally based on the problem.

There are multiple options to use *CHatt* and/or *SPatt* modules, such as using both in parallel, using one of them, using both with *SPatt* first, and using both with *CHatt* first. Based on the experimental findings reported in the main paper of the presented CBAM method, we decided to investigate *CHatt* alone, *SPatt* alone, and CHatt–SPatt (*CHSPatt* (first *CHatt* and then *SPatt*)). As suggested in the main paper, the attention modules are applied either after one of the convolutional layers or after every convolutional layer. In this study, we conducted experiments regarding how to use this attention mechanism from the perspective of resource-constrained devices while using sensor data since they have negligible parameter numbers. In Section 3.2, we explain how we integrate the attention modules into the DeepConvLSTM architecture.

### 3.2. DeepConvLSTM

DeepConvLSTM, presented in [30], is one of the most popular deep learning architectures proposed for sensor-based multimodal wearable activity recognition. Closely related activities/gestures (such as open and close door or drawer) can be distinguished much more effectively using this architecture [45].

As reported in [46], hybrid models that incorporate CNN and LSTM tend to perform better than single models. As the name suggests, DeepConvLSTM architecture combines convolutional and LSTM recurrent layers to extract discriminative features and model temporal dependencies. It achieves state-of-the-art results, and it particularly performs better on recognizing closely related activities, such as *open/close door* [3].The original DeepConvLSTM takes a time window sensor as a 2D input, where width is the sensor signal and height is the time dimension. For example, suppose that there are 3-dimensional accelerometer and gyroscope sensors with a sampling rate of 30 Hz and the window size is selected as 1 second; therefore, the width of the input is 6 (accelerometer x, y, and z; and gyroscope x, y, and z) height is 30 (30×1). Then, the input is passed to the four consecutive convolutional layers with 64 filters and then two consecutive LSTM layers with 128 hidden units. The output of the final LSTM layer is given to a softmax function in order to generate class probabilities of the given input.

In this study, we used the well-known DeepConvLSTM model, which is already capable of modelling temporal dependencies. Moreover, we added CBAM to the existing DeepConvLSTM model to enhance the model’s performance by focusing on important channels and spatial regions within the convolutional layers of the architecture. By using a method focusing on the temporal aspect of sensor-based HAR with improvements in convolutional layers from channel and spatial aspects, we aim to have more accurate and powerful models for sensor-based HAR.

The overall architecture, including DeepConvLSTM with CBAM used in this study, is presented in Figure 1. It can be seen that channel mentioned in *CHatt* reflects the 64 channels of each convolutional layer, and each of them contains the features extracted using convolutions along the temporal axis (height dimension). *CHatt* module helps to rescale the importance of each channel in the input feature. For the *SPatt*, pooling along channels are used, and therefore we achieve one mask for each signal–temporal feature pair. More details about how averaging along different dimensions reflect different properties in time series can be found in [47]. Additionally, note that in the presented architecture, *CHatt* is followed by *SPatt* (*CHSPatt*), which is applied after each convolutional layer of the DeepConvLSTM architecture. However, in our experiments, we applied *CHatt*, *SPatt*, or *CHSPatt* after the first, second, third, fourth, or all convolutional layers. The architecture presented shows us the maximum possible attention applied to the DeepConvLSTM architecture in this study.

### 3.3. Datasets

In this section, we present the two datasets used in this study, which are extensively used in the sensor-based HAR domain [3], mainly when attention mechanism is the focus [19,27,29,34,35,36]. Other reasons to use these datasets include the following: (i) the data are collected from various positions, (ii) the datasets are sufficiently large for taking advantage of using deep learning algorithms, and (iii) there is a diversity in activities performed, including micro activities (e.g., gestures in Opportunity) and daily life activities.

**Figure 2:**
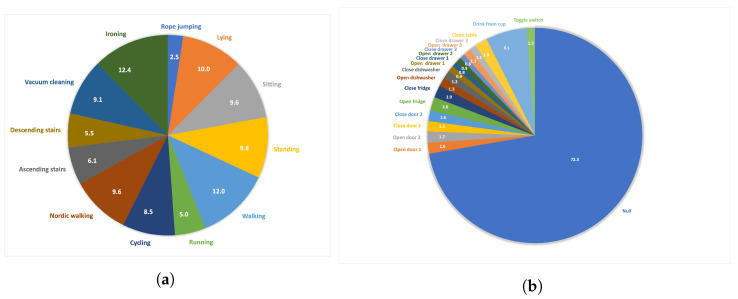
Pie chart of the record distributions of each activities/gestures in the datasets (in %). (**a**) Pamap2; (**b**) Opportunity.

**Physical Activity Monitoring Dataset (Pamap2) [32]** is a benchmark dataset used in multimodal human activity monitoring. The activity data of participants are captured by three inertial measurement units (IMUs) worn on the dominant wrist, chest, and ankle with a sampling rate of 100 Hz, a heart rate sensor with a sampling rate of 9 Hz, and a thermometer. Nine participants were involved in the data collection phase. The dataset contains 18 different daily activities. In this study, we investigated 12 activities that were performed by all participants (the remaining 6 were optional activities, such as watching TV, driving a car, or playing soccer (see Figure 2a for the ratio of activities). Furthermore, since the focus was on capturing the movement of participants using signals coming from different body parts, we used only IMUs located in 3 body parts which resulted in 27 signals for each time step (9 signals for each body part: accelerometer (x, y and z), gyroscope (x, y and z), and magnetometer (x, y and z)). The activities investigated in this study were lying, sitting, standing, walking, running, cycling, Nordic walking, ascending stairs, descending stairs, vacuum cleaning, ironing, and rope jumping.

**Opportunity** [33] includes daily activities and gestures collected by various sensors, such as wearable, object, and ambient sensors. HAR studies mainly focus on data collected using body-worn sensors to classify 4 locomotion and 17 microactivities. Five IMUs were placed on the upper body: left lower arm, left upper arm, right upper arm, right lower arm, and back of the torso. The remaining two sensors were placed on the shoes of users. Since the purpose of this study was to investigate the impact of CBAM on a well-known DeepConvLSTM architectural structure, we only used 5 IMUs on the upper body because they are equivalent IMU devices with similar sensors embedded (3-dimensional accelerometers, gyroscopes, and magnetometers). The gestures performed are open door 1, open door 2, close door 1, close door 2, open fridge, close fridge, open dishwasher, close dishwasher, open drawer 1, close drawer 1, open drawer 2, close drawer 2, open drawer 3, close drawer 3, clean table, drink from a cup, toggle switch, and a null class. The ratio of each gesture in the dataset can be found in Figure 2b. This study focused on gestures rather than the four locomotion activities because the gestures performed have very similar movements, and some of them are very similar to each other, such as opening drawers 1 and 2, which makes them more challenging. The initial sampling rate 30 Hz was used in experiments.

### 3.4. Implementation Details

DeepConvLSTM models with CBAM were implemented using TensorFlow [48] (version 2) and Keras libraries in Python. The Adam optimizer was used in training with its default parameters. The batch size was 64, and the learning rate was 0.001. Since this study was based on a multiclass classification problem, HAR, the categorical cross-entropy loss function, was utilized. Although no overfitting was observed in the analyses performed on the Pamap2 data, an overfitting issue occurred during experiments with the Opportunity dataset. To address this problem, a 0.5 dropout rate was added to each of the two LSTM layers to mitigate overfitting. Additionally, to ensure that the initializations were reproducible for comparing model performances across different runs, the same seed value was used while the deep models’ parameters were being initializing. In addition, 60%, 20%, and 20% of the datasets were used for the training, test, and validation phases, respectively. Five-fold cross validation was also investigated to better understand the proposed models’ reliability. Google Colaboratory [49] using Python (version 3) was used to the conduct experiments presented in this study. Interested readers can email the authors for the source code of the experiments conducted.

## 4. Results

In this section, we present experimental results for activity recognition performance using the DeepConvLSTM network as a backbone with the CBAM method. We first examine channel and spatial attention individually and then in combination. We use macro f1 score (denoted as f1-score), defined as the arithmetic mean of classes’ f1-scores, to reflect the more accurate model performances of models employing imbalanced datasets. Note that for Pamap2, 50 Hz was used as the sampling rate and 30 Hz for Opportunity. Descriptions of abbreviations used in this section for different attention settings are presented in Table 1.

While we aimed to improve recognition performance with the attention mechanisms, it was also crucial to avoid increasing resource consumption of the models since they are expected to be deployed on resource-constrained edge devices such as mobiles and wearables for HAR. The number of parameters in the model directly impacts the computational resources, such as CPU cycles, needed during both the training and testing phases. CPU cycles will also impact the battery consumption of the target devices. In other words, resource consumption and computation time will increase as the number of parameters in the model increases. Therefore, as we examine each attention mechanism in this direction, it is important to consider the number of additional parameters it brings to the model.

Before examining the recognition performance of the models, we first present the number of parameters in each model in Table 2. In our experiments, the resource requirement that the CBAM attention mechanism imposed on the DeepConvLSTM model slightly increased the total number of parameters of the model. However, the number of parameters required for spatial attention was very low compared to that of channel attention. Therefore, in cases where both the channel and spatial attention mechanisms achieve similar recognition performances, it will be more advantageous to select the one that applies spatial attention in terms of resource consumption.

### 4.1. Experiments with Channel Attention

In this section, we discuss the impact of channel attention with the use of various reduction ratios (*r*: 1, 2, 4, 8, and 16) at different depths (such as after layer 1, layer 2, layer 3, and layer 4). As mentioned, abbreviations used for reduction ratios *r* and the number of layers after which attention was applied *x*, are given in Table 1.

Channel attention, as mentioned in Section 3, helps determine which channels are more important and which are confusing/irrelevant in a given input feature. The channel attention parameter *r* is used to determine how complex the *MLP* will be in the module and therefore the number of additional parameters required. As presented in Table 2, in our experiments, *CHatt* requires 8320, 4192, 2128, 1096, and 580 additional parameters for *r*, which is equal to 1, 2, 4, 8, and 16, respectively. In comparing two channel attention experiments with comparable performance results, it is better to decide on the one with a higher *r* value and consequently fewer additional parameters.

#### 4.1.1. Results Using the Train–Test Split

**Figure 3:**
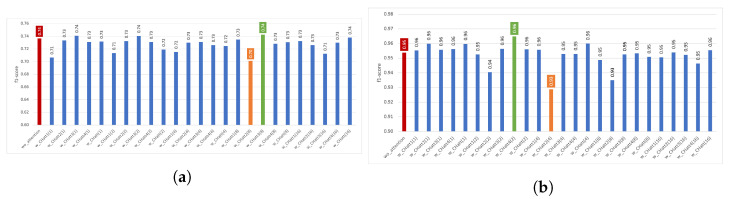
Impact of channel attention on recognition performance using various parameters. (**a**) Impact of *CHatt* on Opportunity, (**b**) Impact of *CHatt* on Pamap2.

In Figure 3, the performance results obtained using channel attention are presented for the two datasets. The red bar represents DeepConvLSTM without attention; the orange bar represents the best performance obtained using *CHatt*, and the green one is the worst performance. For the Pamap2 dataset, the performance results using channel attention range between 0.93 and 0.96, while the performance without attention is 0.95. For the Opportunity dataset, the recognition performance of the wo_attention model is 0.74, while the performances with channel attention vary between 0.70 and 0.74. In addition, adding channel attention after convolutional layer 3 (CHatt3(r)) provides higher recognition performances for all *r* values, except for the case where *r* is 16. The lowest results are usually obtained when channel attention is applied just after the first convolutional layer (CHatt1(r)).

**Figure 4:**
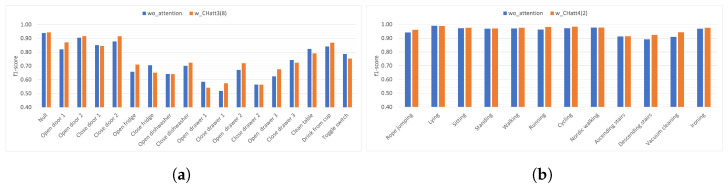
Gesture/Activity based example: impact of channel attention. (**a**) w_CHatt3(8) using Opportunity dataset, (**b**) w_CHatt4(2) using Pamap2 dataset.

In Figure 4a gesture-based performance results without attention and with best-performing channel attention setting (CHatt3(8)) for the Opportunity dataset are presented. We can observe that in some gesture pairs that are frequently confused with each other, the performance of one increases while the other decreases at a similar rate after the channel attention is applied, such as *Open/Close fridge* or *Open/Close drawer 1*. More clearly, after attention is applied, the performance of *Open fridge* increases by 0.05 (from 0.66 to 0.71), while the performance of Close fridge decreases by 0.06 (from 0.71 to 0.65). Similarly, when attention is applied, *Close drawer 1* increases by 0.05 (0.52 to 0.57), while *Open drawer 1* decreases by 0.05 (from 0.59 to 0.54). Considering the remaining gestures, we can observe that the performance of seven of them improve over 0.01, and three of them decrease by over 0.01. Moreover, for the Pamap2 dataset, Figure 4b shows the recognition performance results for each activity, both without using attention and with channel attention applied to the fourth convolutional layer with a reduction ratio of two (CHatt4(2)) as an example. For activities whose performance is already above 0.95, attention does not provide a significant improvement. However, *Descending stairs*, which initially has the lowest performance without attention, reaches a performance increase with the use of the channel attention module (from 0.89 to 0.92). Similarly, the activity *Vacuum cleaning*, which has the second lowest performance, achieved the highest performance increase (from 0.91 to 0.94) with the use of attention. Overall, for the Pamap2 dataset, we can say that channel attention brings a slight improvement in the average recognition performance. However, for the mostly confused activities, using attention increases the performance by 0.02.

#### 4.1.2. Results Using Five-Fold Cross Validation

**Figure 5:**
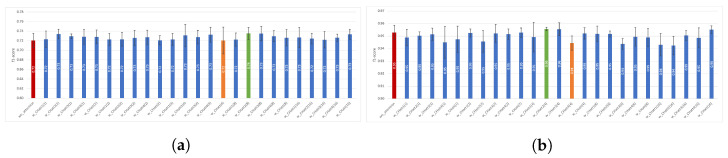
Impact of channel attention on recognition in terms of performance and sstd using various parameters. (**a**) Impact of *CHatt* on Opportunity, (**b**) Impact of *CHatt* on Pamap2.

We also investigated channel attention’s impact using a five-fold cross validation technique. We separately reported each experiment’s average macro f1-score (denoted as f1-score) and sample standard deviation (sstd) of folds. In Figure 5, the effects of channel attention on the Opportunity and Pamap2 datasets are presented. For the Opportunity dataset, we can observe that the model’s performance improved in all experiments with channel attention, except for one case (*w_CHatt(4)*), which obtained equal performance with *wo_attention*. The highest increase of 0.02 (from 0.72 to 0.74) can be observed for *w_CHatt2(8)*. Moreover, *w_CHat3(1)* achieved the lowest sstd of 0.005, which was 0.015 with *wo_attention*. As for the Pamap2 dataset, there was no critical increase in f1-score; however, by using channel attention models, we obtained more robust models, as with *w_CHatt2(4)* (sstd of 0.001), which was higher using *wo_attention* (sstd of 0.006).

### 4.2. Experiments with Spatial Attention

In this section, we discuss how spatial attention impacts recognition performance in relation to the use of various parameters (*r*: 3, 5, and 7) at different depths.

#### 4.2.1. Results Using Train–Test Split

**Figure 6:**
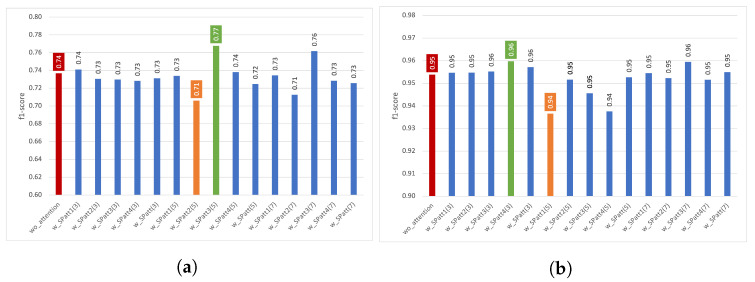
Impact of spatial attention on recognition performance using various settings. (**a**) Impact of *SPatt* on Opportunity; (**b**) Impact of *SPatt* on Pamap2.

In Figure 6, the results of different spatial attention experiments are presented. For the Pamap2 dataset, applying spatial attention did not bring a significant performance increase. However, for the Opportunity dataset, after using w_SPatt3(5), we observed an increase of around 0.03 (from 0.74 to 0.77).

It can observed that when the model has a good activity recognition capability, as in the case of the Pamap2 dataset with a performance of around 0.95, the attention mechanisms do not significantly impact the performance. However, we can observe an improvement in the Opportunity dataset. In Opportunity, activities are more challenging to classify, and the dataset is not balanced because 72% the of data comes from the Null class; therefore, the overall recognition performance values are low (around 0.70) compared to those of Pamap2.

Furthermore, for the Opportunity dataset, the spatial attention results are better than the channel attention results. It should be noted that the number of additional parameters required for spatial attention is far lower than that of channel attention (see Table 2 in Section 4). Therefore, using spatial attention also has an advantage in terms of the number of parameters required for the Opportunity dataset. However, for the Pamap2, we did not observe a major performance increase with either spatial attention or channel attention.

**Figure 7:**
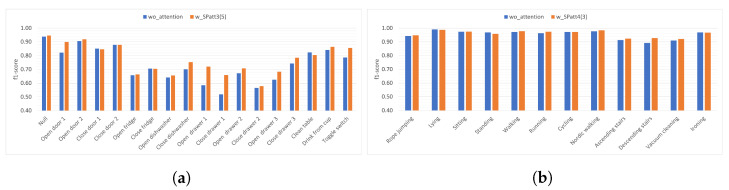
Gesture/Activity based example: impact of spatial attention. (**a**) w_SPatt3(5) using Opportunity dataset, (**b**) w_SPatt4(3) using Pamap2 dataset.

Figure 7a presents the f1-scores for each gesture in the Opportunity dataset. We can observe that the highest performance increases from spatial attention after the third convolutional layer with a kernel size of 5 (SPatt3(5)) are obtained for *Close drawer 1* (from 0.52 to 0.66) and *Open drawer 1* (from 0.59 to 0.72), which are the two activities with the worst performance when attention is not used. Although the recognition performance of most gestures is positively affected by the attention mechanism, *Clean table* gesture is an exception in this analysis and is negatively affected (with a decrease of 0.02) from the application of attention. Considering the activity-based results presented in Figure 7b for the Pamap2 dataset, which are similar to those we obtained in Figure 4b, we once again can observe the highest increases in the lowest-performing activities, which are *Ascending stairs*, *Descending stairs*, and *Vacuum cleaning* with a performance increase of 0.01, 0.04, and 0.01, respectively. Overall, spatial attention improves performance by, on average, 0.02 for Pamap2’s three worst-performing activities (lower than 0.92 in the wo_attention setting) and by, on average, 0.10 for Opportunity’s three worst=performing gestures (lower than 0.60 in the wo_attention setting).

#### 4.2.2. Results Using Five-Fold Cross Validation

**Figure 8:**
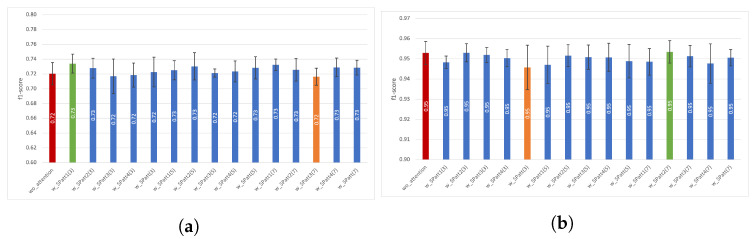
Impact of spatial attention on recognition in terms of performance and sstd using various parameters. (**a**) Impact of *SPatt* on Opportunity, (**b**) Impact of *SPatt* on Pamap2.

The results of spatial attention experiments using five-fold cross validation are presented in terms of f1-score and sstd in Figure 8 for Opportunity and for Pamap2. The use of spatial attention obtained the highest increases with *w_SPatt1(3)* (from 0.72 to 0.73) for Opportunity. Moreover, adding spatial attention could help obtain similar f1-score performances with more robust models. For example, instead of selecting the *wo_attention* model, which has an f1-score of 0.72 and an sstd of 0.015, we can choose *w_SPatt3(5)*, which has an f1-score of 0.72 and an sstd of 0.006. Therefore, this model is more robust and can provide more reliable results.

### 4.3. Experiments with Channel–Spatial Attention

In this section, we discuss the impact of applying channel attention first and then spatial attention together. In this analysis, we used combinations of different channel and spatial attention parameters at different depths.

#### 4.3.1. Results Using Train–Test Split

**Figure 9:**
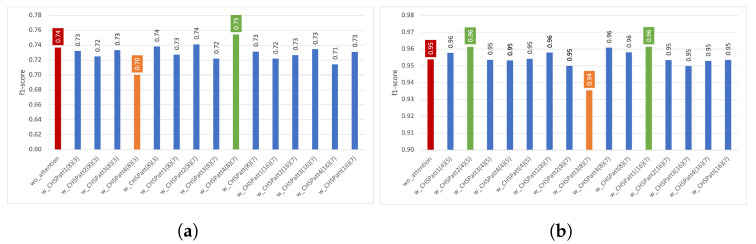
Impact of channel-spatial attention on recognition performance using various settings. (**a**) Impact of *CHSPatt* on Opportunity, (**b**) Impact of *CHSPatt* on Pamap2.

The performance results for the Opportunity and Pamap2 datasets are presented in Figure 9. Considering the Opportunity dataset, we can observe that the best average performance using channel–spatial attention is obtained for w_CHSPatt4(8)(7), which is 0.75. This value is higher than the best result obtained with only channel attention (0.74 with w_CHatt3(8)) and lower than the best result obtained with only spatial attention (0.77 with w_SPatt3(5)). We can conclude that using a combination of attention mechanisms does not provide the best result and that only one (such as channel attention) may suffice to achieve higher results than those with channel–spatial attention. Furthermore, the best performance obtained using channel–spatial attention is not significantly higher than the result obtained without attention (an increase from 0.95 to 0.96). We present the results obtained for the most successful and least successful CHSPatt settings for ease of reading. However, the results for all CHSPatt experiments are also presented in the Appendix A, Table A1.

#### 4.3.2. Results Using Five-Fold Cross Validation

**Figure 10:**
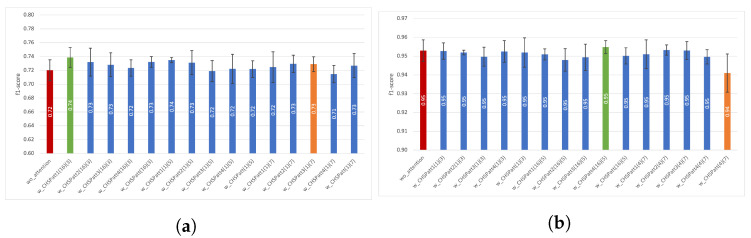
Impact of channel-spatial attention on recognition in terms of performance and sstd using various parameters. (**a**) Impact of *CHSPatt* on Opportunity; (**b**) Impact of *CHSPatt* on Pamap2.

For the impact of adding both attention mechanisms, the results obtained for the best- and worst-performing experiments compared to the performance of *wo_attention* are presented in Figure 10 for both datasets. For Opportunity, compared to *wo_attention*, we can observe that the greatest improvement in recognition performance was obtained with *w_CHSPatt1(16)(3)* (from an f1-score of 0.72 to 0.74 and from 0.015 sstd to 0.014 sstd). However, to have a more robust model, one may also choose an attention module with less or no improvement but more robustness. For Opportunity, this model could be *w_CHSPatt1(1)(5)* with an f1-score of 0.73 and 0.004 sstd, while for Pamap2, this could be an f1-score of *w_CHSPatt2(1)(3)* (0.95 and 0.001 sstd).

**Figure 11:**
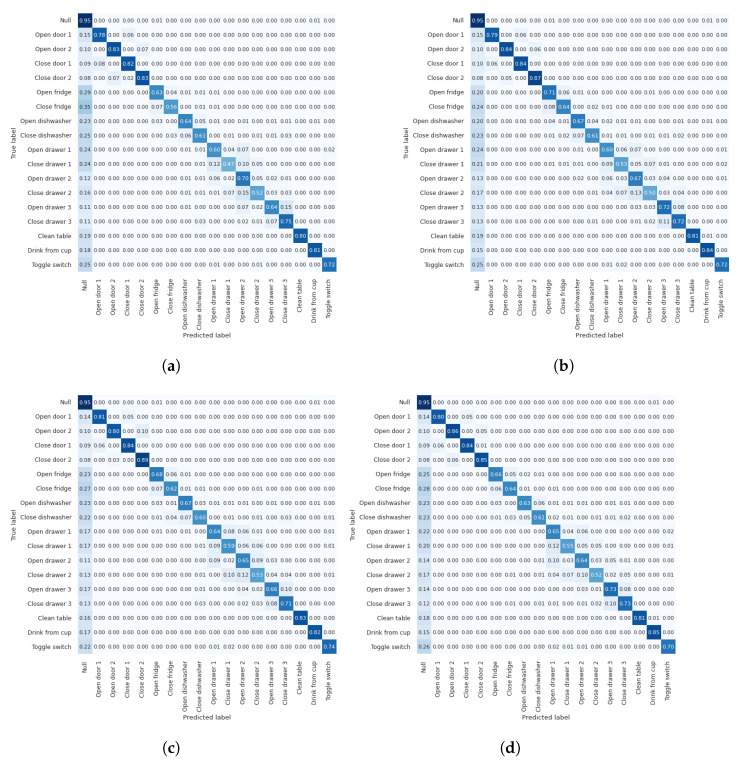
Confusion matrices of using best-performing models of channel, spatial and channel-spatial models and without attention model for Opportunity dataset. (**a**) *wo_attention* (average f1-score is 72.04%), (**b**) *w_CHatt2(8)* (average f1-score is 73.52%), (**c**) *w_SPatt1(3)* (average f1-score is 73.39%), (**d**) *w_CHSPatt1(16)(3)* (average f1-score is 73.88%).

**Figure 12:**
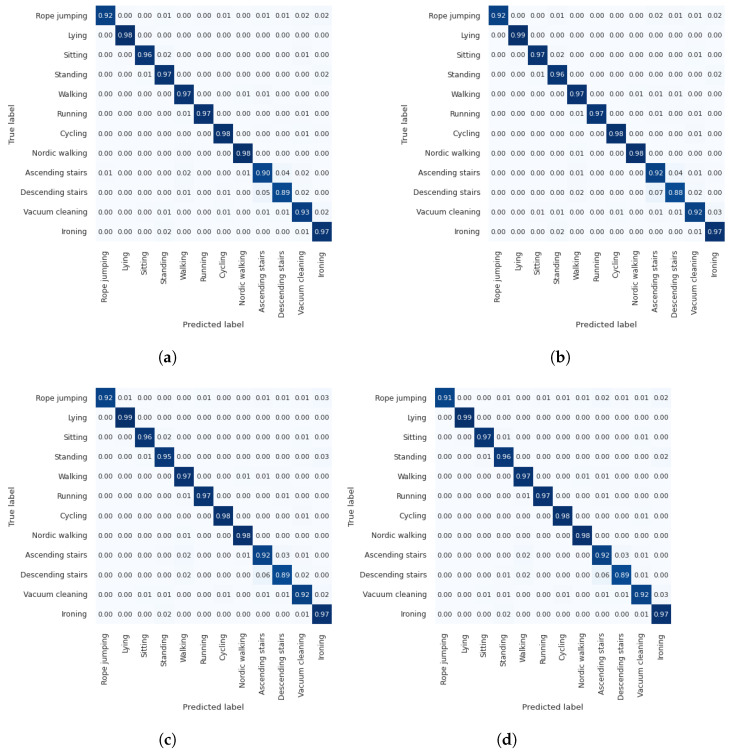
Confusion matrices of using best-performing models of channel, spatial and channel-spatial models and without attention model for Pamap2 dataset. (**a**) *wo_attention* (average f1-score is 0.95), (**b**) *w_CHatt2(4)* (average f1-score is 0.96), (**c**) *w_SPatt2(7)* (average f1-score is 0.95), (**d**) *w_CHSPatt4(16)(5)* (average f1-score is 0.95).

We investigated the impact of attention on each activity/gesture in the dataset by comparing the *wo_attention* model with the best-performing models of channel, spatial, and channel–spatial attention modules for both datasets. Figure 11d and Figure 12d present the combined confusion matrices for *wo_attention* and several attention modules for Opportunity and Pamap2, respectively.

In Figure 11, we can observe from the confusion matrices for the Opportunity dataset that almost all gestures are most frequently mispredicted as the Null class. The Null class constitutes more than 70% of the data. Apart from this, the highest confusion is apparent between drawer-related activities (e.g., 0.15 of *Close drawer 2* samples are falsely predicted as *Open drawer 2*, and 0.15 of *Close drawer 3* samples are falsely predicted as *Open drawer 3). The Clean table, Drink from a cup, and Toggle switch* gestures perform better than do the rest. More particularly, their confusion apart from the *Null* class remains under 0.02. The gestures with the worst performance in the *wo_attention* model, where only 0.47, 0.52, and 0.56 of the samples are correctly predicted, are *Close drawer 1*, *Close drawer 2*, and *Close fridge*, respectively. Owing to the attention modules, a significant 0.12 and 0.08 improvement is achieved for the *Close drawer 1* and *Close fridge* gestures, respectively, while this improvement is only 0.01 for *Close drawer 2*. Moreover, when the confusion matrices of Pamap2 datasets were investigated (presented in Figure 12), a significant improvement could not be observed in the activity-based performance, which is similar to the attention mechanism, contributing an average f1-score of less than 0.01. However, as we mentioned previously, the attention contributes to the model’s robustness, even if there is no significant increase in performance. Finally, to determine if baseline models with attention-applied models were statistically significant from the baseline models without attention (wo_attention), we conducted one-sample *t*-tests for each of the Opportunity and Pamap2 datasets. There were two input samples used, one for each dataset. For both datasets, input samples provided to the statistical tests included 1 performance result obtained with the baseline model (wo_attention) and 115 performance results obtained with attention-applied models (115 performance results included 5 × 5, 3 × 5, and 15 × 5 performance results derived from channel, spatial, and channel–spatial attention experiments, respectively). Statistical test results showed that attention-applied models achieved significant results when compared against the baseline models without attention for the Opportunity (*p* < 0.001 from Student *t*-test) and Pamap2 datasets (*p* < 0.001 from Wilcoxon *t*-test).

Finally, to determine if baseline models with attention-applied models are statistically significant when compared against baseline without attention models (*wo_attention*), we conducted one-sample *t*-tests for each of the Opportunity and Pamap2 datasets. There are two input samples used, one for each dataset. For both datasets, input samples provided to the statistical tests include one performance result obtained with the baseline model (*wo_attention*) and 115 performance results obtained with attention-applied models (115 performance results have 5 × 5, 3 × 5, and 15 × 5 performance results coming from channel, spatial, and channel-spatial attention experiments, respectively). Statistical test results show that attention-applied models achieve significant results when compared against the baseline without attention model for Opportunity (*p* < 0.001 from Student *t*-test) and Pamap2 datasets (*p* < 0.001 from Wilcoxon *t*-test).

## 5. Discussion

Human activity recognition using multiple sensor data from different body parts is a challenging task. In this study, we, for the first time, applied CBAM to the state-of-the-art sensor-based HAR architecture DeepConvLSTM. While doing this, we investigated the impact of channel and spatial attention modules individually using different parameters (reduction ratio and kernel sizes) and in combination to understand the impact better. By adding channel and spatial attention modules to the DeepConvLSTM model, which is already good for modelling temporal dependencies in time series, we also better captured both channel-wise and spatial patterns in convolutional layers since the CBAM method is easy to apply in any convolutional layer with a negligible number of additional parameters.

The proposed model was evaluated on two public sensor-based HAR datasets: Pamap2 and Opportunity. Pamap2 contains daily life activities such as *sitting and standing*, while Opportunity contains more confusing microactivities/gestures such as *open drawer or close door*. DeepConvLSTM with CBAM outperformed regular DeepConvLSTM with use of channel attention (*CHatt4(2)*) for Pamap2 (from 0.95 to 0.96) and with use of spatial attention (*SP*att3(5)) for Opportunity (from 0.74 to 0.77). Moreover, the number of parameters in the model created for Opportunity increased by about 0.006% with the addition of spatial attention, while the parameter number of Pamap2 increased by about 0.296% with the addition of Channel attention, which indicates this attention method is suitable for resource-constrained environments. Even though DeepConvLSTM is particularly good at discriminating closely related activities (such as *ascending/descending stairs*), we observed that by applying CBAM attention, we particularly improved the performance of the lowest-performing activities. Therefore, we understand that the CBAM improved the feature extraction capability of convolutional layers in DeepConvLSTM. Note that since the CBAM is designed to apply convolutional layers, we could also experiment on a CNN network; however, since hybrid models (CNN+LSTM) perform better than does CNN alone and because DeepConvLSTM models also include the temporal aspect of sensor data, we did not experiment with CNN alone.

Finally, we also compared our proposed system with recent deep learning architectures that employ attention mechanisms in the sensor-based HAR domain on the same datasets. The performance results with the number of parameters (when available) are presented in Table 3. We can observe that the number of parameters is high for both datasets in [27] compared to the number of parameters that we need in our system. We could not make a fair comparison since accuracy was provided as the performance metric and the datasets were imbalanced, with Opportunity being especially y imbalanced. Considering [25], we can only compare the Pamap2 results, and we observed that the number of parameters is low, but the recognition performance was not higher than our study. In [19], where attention in the temporal context was considered, the authors achieved a significant improvement over what they obtained without attention. However, comparing the final results, we found that we could achieve higher scores.

## 6. Conclusions

In this study, we investigated sensor-based human activity recognition with channel and spatial attention modules of the convolutional block attention module (CBAM). We followed a detailed analysis, with a focus on channel attention, spatial attention, and channel–spatial attention. We used a hybrid sensor-based HAR model, DeepConvLSTM, and applied attention at different depths to its convolutional layers. We evaluated the impact of models using two HAR datasets: Pamap2 and Opportunity. We observed that for each channel, spatial, and channel–spatial attention, applying attention outperformed the model’s initial (without attention) performance. Moreover, the highest increase was observed using *CHatt4(2)* (from 0.95 to 0.96) for Pamap2 and using *SPatt3(5)* (from 0.74 to 0.77) for Opportunity. Since the activities in the Pamap2 dataset are not as challenging as those in Opportunity, the initial DeepConvLSTM performance was lower in Opportunity. Thus, the effect of attention was more evident.

## Figures and Tables

**Table 1:** Description of abbreviations used to define the attention setting used in the experiments.

Abbreviation	Description
w	With
wo	Without
CHattx(r)	Channel attention module applied to $x^{th}$ convolutional layer with a reduction ratio *r*
CHatt(r)	Channel attention modules applied to all four convolutional layers with a reduction ratio *r*
SPattx(k)	Spatial attention module applied to $x^{th}$ convolutional layer with a kernel size *k*
SPatt(k)	Spatial attention modules applied to all four convolutional layers with a kernel size *k*
CHSPattx(r)(k)	First channel and then spatial attention module (CHSPatt or CBAM) applied to $x^{th}$ convolutional layer with a reduction ratio *r* and kernel size *k* for channel and spatial attention, respectively
CHSPatt(r)(k)	First channel and then spatial attention module (CHSPatt or CBAM) applied to all four convolutional layers with a reduction ratio *r* and kernel size *k* for channel and spatial attention, respectively

**Table 2:** Comparison of the number of additional parameters required in the experiments. The base models (wo_attention) require 822,098 parameters for Opportunity and 1,415,244 parameters for Pamap2.

	Reduction Ratio (r)	Kernel Size (k)	# of Additional Parameters
	1		8320
	2		4192
w_CHattx(r)	4		2128
	8		1096
	16		580
		3	18
w_SPattx(k)		5	50
		7	98
		3	8338
	1	5	8370
		7	8418
		3	4210
	2	5	4242
		7	4290
		3	2146
w_CHSPatt(r)(k)	4	5	2178
		7	2226
		3	1114
	8	5	1146
		7	1194
		3	598
	16	5	630
		7	678

**Table 3:** Performance comparison of existing models on Opportunity and Pamap2 datasets.

		Opportunity		Pamap2	
		Performance Result	# of Parameters	Performance Result	# of Parameters
This study	DeepConvLSTM	0.74 of f1-score	0.82 M	0.95 of f1-score	1.42 M
	DeepConvLSTM + CBAM Attention	0.77 of f1-score	0.82 M	0.96 of f1-score	1.42 M
[19]	DeepConvLSTM	0.67 of f1-score		0.75 of f1-score	
	DeepConvLSTM + Temporal Attention in LSTM	0.71 of f1-score		0.87 of f1-score	
[27]	CNN	0.78 of accuracy	1.15 M	0.91 of accuracy	2.73 M
	CNN + BlockAttention	0.80 of accuracy	1.17 M	0.92 of accuracy	2.75 M
[25]	CNN			0.91 of f1 score	0.86 M
	CNN + Temporal, Channel, Spatial			0.92 of f1 score	0.86 M

## Data Availability

Pamap2 and Opportunity datasets that support the findings of this study are openly available in “UCI Machine Learning Repository” at https://archive.ics.uci.edu/ml/datasets/pamap2+physical+activity+monitoring accessed on 24 May 2023 and https://archive.ics.uci.edu/ml/datasets/opportunity+activity+recognition, accessed on 24 May 2023, respectively.

## Appendix A. Additional Results

**Table A1:** The f1-score performance results of all CHSPatt experiments for Opportunity and Pamap2 datasets.

	Opportunity	Pamap2	(Continue)	Opportunity	Pamap2
wo_attention	0.74	0.95			
w_CHSPatt1(1)(3)	0.72	0.95	w_CHSPatt1(8)(5)	0.71	0.95
w_CHSPatt2(1)(3)	0.73	0.95	w_CHSPatt2(8)(5)	0.73	0.96
w_CHSPatt3(1)(3)	0.73	0.96	w_CHSPatt3(8)(5)	0.73	0.95
w_CHSPatt4(1)(3)	0.73	0.96	w_CHSPatt4(8)(5)	0.74	0.95
w_CHSPatt(1)(3)	0.73	0.95	w_CHSPatt(8)(5)	0.72	0.96
w_CHSPatt1(2)(3)	0.73	0.96	w_CHSPatt1(16)(5)	0.72	0.96
w_CHSPatt2(2)(3)	0.73	0.95	w_CHSPatt2(16)(5)	0.71	0.96
w_CHSPatt3(2)(3)	0.72	0.96	w_CHSPatt3(16)(5)	0.73	0.95
w_CHSPatt4(2)(3)	0.73	0.95	w_CHSPatt4(16)(5)	0.75	0.96
w_CHSPatt(2)(3)	0.73	0.94	w_CHSPatt(16)(5)	0.72	0.95
w_CHSPatt1(4)(3)	0.75	0.96	w_CHSPatt1(1)(7)	0.74	0.96
w_CHSPatt2(4)(3)	0.73	0.96	w_CHSPatt2(1)(7)	0.72	0.95
w_CHSPatt3(4)(3)	0.72	0.96	w_CHSPatt3(1)(7)	0.73	0.95
w_CHSPatt4(4)(3)	0.73	0.95	w_CHSPatt4(1)(7)	0.72	0.95
w_CHSPatt(4)(3)	0.72	0.96	w_CHSPatt(1)(7)	0.71	0.95
w_CHSPatt1(8)(3)	0.73	0.95	w_CHSPatt1(2)(7)	0.73	0.96
w_CHSPatt2(8)(3)	0.72	0.95	w_CHSPatt2(2)(7)	0.70	0.96
w_CHSPatt3(8)(3)	0.73	0.96	w_CHSPatt3(2)(7)	0.73	0.94
w_CHSPatt4(8)(3)	0.70	0.95	w_CHSPatt4(2)(7)	0.73	0.96
w_CHSPatt(8)(3)	0.74	0.95	w_CHSPatt(2)(7)	0.73	0.96
w_CHSPatt1(16)(3)	0.73	0.95	w_CHSPatt1(4)(7)	0.72	0.94
w_CHSPatt2(16)(3)	0.71	0.95	w_CHSPatt2(4)(7)	0.71	0.96
w_CHSPatt3(16)(3)	0.73	0.96	w_CHSPatt3(4)(7)	0.75	0.95
w_CHSPatt4(16)(3)	0.74	0.96	w_CHSPatt4(4)(7)	0.73	0.95
w_CHSPatt(16)(3)	0.72	0.95	w_CHSPatt(4)(7)	0.73	0.96
w_CHSPatt1(1)(5)	0.73	0.95	w_CHSPatt1(8)(7)	0.73	0.96
w_CHSPatt2(1)(5)	0.73	0.94	w_CHSPatt2(8)(7)	0.74	0.95
w_CHSPatt3(1)(5)	0.73	0.95	w_CHSPatt3(8)(7)	0.72	0.94
w_CHSPatt4(1)(5)	0.75	0.96	w_CHSPatt4(8)(7)	0.75	0.96
w_CHSPatt(1)(5)	0.72	0.96	w_CHSPatt(8)(7)	0.73	0.96
w_CHSPatt1(2)(5)	0.73	0.96	w_CHSPatt1(16)(7)	0.72	0.96
w_CHSPatt2(2)(5)	0.73	0.96	w_CHSPatt2(16)(7)	0.73	0.95
w_CHSPatt3(2)(5)	0.72	0.96	w_CHSPatt3(16)(7)	0.73	0.95
w_CHSPatt4(2)(5)	0.72	0.96	w_CHSPatt4(16)(7)	0.71	0.95
w_CHSPatt(2)(5)	0.73	0.95	w_CHSPatt(16)(7)	0.73	0.95
w_CHSPatt1(4)(5)	0.71	0.96			
w_CHSPatt2(4)(5)	0.73	0.96			
w_CHSPatt3(4)(5)	0.73	0.95			
w_CHSPatt4(4)(5)	0.75	0.95			
w_CHSPatt(4)(5)	0.72	0.95			
